# FOXA2 as a SETD1A-Regulated Driver of Tamoxifen Resistance in Breast Cancer

**DOI:** 10.32604/or.2025.072592

**Published:** 2026-02-24

**Authors:** Myeong Ryeo Kim, Jae Rim Lee, Xiaohan Zhang, Kwang Won Jeong

**Affiliations:** 1 Gachon Institute of Pharmaceutical Sciences, College of Pharmacy, Gachon University, 191 Hambakmoero, Yeonsu-gu, Incheon, 21936, Republic of Korea; 2 School of Pharmaceutical Sciences, Guizhou University, Guiyang, 550025, China

**Keywords:** Tamoxifen resistance, forkhead box protein A2, SET domain containing 1A, breast cancer, cancer stem cells

## Abstract

**Objectives:**

Tamoxifen is a key drug that provides endocrine therapy for estrogen receptor (ER) α-positive breast cancer; however, resistance remains a significant clinical challenge. This study aims to investigate the molecular mechanisms of tamoxifen resistance in ERα-positive breast cancer, with particular focus on the role of SET Domain Containing 1A (SETD1A)-driven forkhead box A2 (FOXA2) as a key regulator of this resistance.

**Methods:**

FOXA2 expression and its regulation by SETD1A were assessed via (quantitative polymerase chain reaction), western blotting, transcriptome profiling, and chromatin immunoprecipitation analyses. The effects of FOXA2 on cell proliferation, migration, invasion, and cancer stem cell traits were evaluated using small interfering RNA (siRNA)-mediated silencing. Clinical relevance was examined by analyzing patient datasets and tumor tissue microarrays.

**Results:**

FOXA2 expression was significantly elevated in tamoxifen-resistant (TamR) and ERα-negative breast cancer cells compared to that in ERα-positive MCF-7 cells, regardless of tamoxifen treatment or ERα depletion. Transcriptome and chromatin immunoprecipitation analyses revealed that SETD1A, a histone methyltransferase, directly regulated FOXA2 expression. Functionally, FOXA2 knockdown inhibited the proliferation, migration, invasion, and cancer stem cell properties of TamR cells while restoring tamoxifen sensitivity. High FOXA2 expression was correlated with poor survival and reduced responsiveness to tamoxifen in patients with ER-positive breast cancer.

**Conclusion:**

Our findings identified FOXA2 as a key mediator of tamoxifen resistance regulated by SETD1A and suggested that targeting the SETD1A-FOXA2 axis may offer a novel strategy for overcoming endocrine resistance in breast cancer.

## 1 Introduction

Tamoxifen is an anti-estrogen medication that blocks estrogen receptors in breast cancer (BC) cells, thereby inhibiting cancer cell growth [1]. Tamoxifen resistance in BC refers to the phenomenon wherein BC cells become insensitive or resistant to the effects of tamoxifen, a commonly used hormone therapy drug. BC cells can develop resistance to tamoxifen through several mechanisms, including estrogen receptor (ER) mutations, activation of alternative signaling pathways, increased growth factor signaling, alterations in cellular apoptotic pathways, metabolic changes, and microenvironmental factors [2–4]. Managing tamoxifen resistance often involves switching to other types of hormone therapies or combining alternative therapies to overcome resistance mechanisms [5]. Current research on these mechanisms focuses on developing new strategies and drugs to combat tamoxifen resistance and improve outcomes in patients with hormone receptor-positive BC.

The Forkhead box A (FOXA) gene family, including FOXA1, FOXA2, and FOXA3, comprises transcription factors crucial for embryonic development, immune regulation, cell cycles, longevity, and energy metabolism [6–8]. FOXA1 is a pioneer factor because it binds to condensed chromatin and facilitates the recruitment of ERα to estrogen response elements (EREs) in the DNA [9,10]. High FOXA1 expression is associated with better clinical outcomes in ERα-positive BCs, as it indicates intact ERα signaling and potential responsiveness to endocrine therapies such as tamoxifen and aromatase inhibitors [11,12]. FOXA2, also known as hepatocyte nuclear factor 3-β (HNF3B), shares 95% protein homology with FOXA1 within the forkhead box DNA domain [13]. Although FOXA2 is widely expressed across tissues [14–16], its dysregulated expression is implicated in several key oncogenic processes, including cell proliferation, metastasis, and the maintenance of stemness, across various tumor types such as colorectal cancer (CRC), renal cell carcinoma (RCC), neuroendocrine prostate cancer (NEPC), lung cancer, and ovarian cancer [17–21]. FOXA2 is also commonly upregulated in human RCC samples and promotes RCC proliferation by transcriptionally activating the expression of Hypoxia-Inducible Factor 2 alpha (HIF2α) [18]. In prostate cancer, the transition from adenocarcinoma to the neuroendocrine lineage represents a mechanism of resistance to targeted therapies. FOXA2 cooperates with mutant Kirsten rat sarcoma virus (KRAS) to drive lung invasive mucinous adenocarcinoma [20]. However, the role of FOXA2 in cancer cell resistance of BC and BC stem cells has not been studied in detail.

SET Domain Containing 1A (SETD1A), which belongs to the SET domain family of histone methyltransferases, plays a crucial role in regulating gene expression through histone modification, specifically by methylating histone H3 at lysine 4 (H3K4) [22]. This modification, particularly H3K4 trimethylation (H3K4me3), is associated with active transcription and essential for various biological processes, including development and cancer progression [23,24]. Aberrant expression of SETD1A has been implicated in several human diseases, including acute myeloid leukemia (AML), CRC, and triple-negative BC (TNBC) [25–27]. In this context, dysregulated SETD1A activity contributes to oncogenic processes, such as enhanced cell proliferation and survival. Recent studies have highlighted the role of SETD1A in the survival of tamoxifen-resistant (TamR) BC cells by regulating the expression of non-ERα target genes, suggesting its significance in endocrine therapy resistance mechanisms [27–30]. Notably, it induces the development of TamR BC cells by activating the growth of cancer stem cells (CSCs) via the SETD1A-SOX2 axis [3,31].

In this study, we aim to investigate whether FOXA2 expression differs between ERα-positive and TamR BC cells, and to determine the functional role of FOXA2 in TamR BC, particularly in cell growth, metastasis, and therapeutic response. We further seek to explore whether SETD1A regulates FOXA2 expression during the development of tamoxifen resistance. Through this research, we intend to elucidate the significance of the SETD1A-FOXA2 regulatory axis and assess its potential as a therapeutic target to overcome endocrine resistance in BC.

## 2 Materials and Methods

### 2.1 Cell Culture, Reagents, and Plasmids

MCF-7 (cat# 30022) and MDA-MB-231 (cat# 30026) cell lines were sourced from the Korean Cell Line Bank (KCLB, Seoul, Republic of Korea). The MCF-7 cells used in this study were confirmed to be mycoplasma-free, and their cell identity was verified by DNA fingerprinting analysis using short tandem repeat (STR) markers provided by the KCLB. MCF-7 cells were maintained in high-glucose Dulbecco’s modified Eagle’s medium (DMEM) (Hyclone, cat# SH30243.01, Marlborough, MA, USA) supplemented with 10% fetal bovine serum (FBS) (Gibco, cat# A5256701, Waltham, MA, USA). MDA-MB-231 cells were cultured in a Roswell Park Memorial Institute (RPMI)-1640 medium (Hyclone, cat# SH30027.01) supplemented with 10% FBS. Cell cultures were incubated at 37°C in a humidified environment with 5% CO_2_. To generate TamR cells, MCF-7 cells were subjected to a 12-month treatment regimen with 5 μM 4-hydroxytamoxifen (Tamoxifen) (Sigma-Aldrich, cat# H6278, Darmstadt, Germany) [28]. Full-length FOXA2 (ID# 153110) and SOX2 (ID# 16577) expression plasmids were obtained from Addgene (Watertown, MA, USA). The pcDNA3-flag-SETD1A plasmid was provided by Professor David Skalnik from Purdue University.

### 2.2 Immunoblotting and Immunocytochemistry

Western blotting was performed as previously described [28]. Total protein was extracted using SDS (Sodium Dodecyl Sulfate) lysis buffer. The protein was resolved on SDS-PAGE (8%) and transferred onto PVDF (polyvinylidene fluoride) membranes (Bio-Rad Laboratories, cat# 1620177, Hercules, CA, USA) Blocking of the membranes was performed using 5% bovine serum albumin (BSA, CELLNEST, cat# CNB104-0100, Hanam-si, Republic of Korea) solution for 2 h and incubated overnight at 4°C using an anti-SETD1A antibody (Bethyl Laboratories, cat# A300-289A, Montgomery, AL, USA), FOXA2 (Abclonal Technology, cat# A19053, Woburn, MA, USA), FOXA1(Active Motif, cat# 39837, Carlsbad, CA, USA), ERα (Santa Cruz Biotechnology, cat# SC-514857, Dallas, TX, USA), SOX2 (Thermo Fisher Scientific, cat# MA1-014, Waltham, MA USA), β-actin (Santa Cruz Biotechnology, cat# SC-47778). Antibodies for Western blotting are summarized in Table 1. Subsequently, each membrane was rinsed three times with Tris-buffered saline-Tween (TBST) buffer, with each wash lasting 15 min. The secondary antibodies (1:1000 dilution) used were anti-mouse (Santa Cruz Biotechnology, cat# sc-516102) and anti-rabbit (Cell Signaling Technology, cat# 7074S, Danvers, MA, USA), which were incubated with the membrane for 2 h at room temperature. After washing, the protein bands were visualized using an Immobilon Western Chemiluminescent HRP Substrate (Merck Millipore, cat# WBKLS0500, Burlington, MA, USA) and detected with a ChemiDoc MP imaging system (Bio-Rad Laboratories).

**Table 1:** Antibodies used for western blotting, immunocytochemistry, and Chromatin immunoprecipitation assays

Antibody	Dilution Ratios	Company, City, Province/State, Country	Catalog Number
FOXA2	1:1000	ABclonal (Woburn, MA, USA)	A19053
SETD1A	1:1000	Bethyl Laboratories (Montgomery, AL, USA)	A300-289A
SETD1A	1:100	Santa Cruz Biotechnology (Dallas, TX, USA)	sc-515590
FOXA1	1:1000	Active Motif (Carlsbad, CA, USA)	39837
SOX2	1:1000	Thermo Fisher Scientific (Waltham, MA USA)	MA1-014
ERα	1:200	Santa Cruz Biotechnology (Dallas, TX, USA)	sc-514857
β-actin	1:1000	Santa Cruz Biotechnology (Dallas, TX, USA)	sc-47778
H3K4me3	1:1000	Active Motif (Carlsbad, CA, USA)	39159
Pol II	1:1000	Merck Millipore (Burlington, MA, USA)	05-623
Rabbit IgG	1:1000	Cell Signaling Technology (Danvers, MA, USA)	2729
DyLight 488	1:200	Vector Laboratories (Burlingame, CA, USA)	DI-1488
DyLight 594	1:200	Vector Laboratories (Burlingame, CA, USA)	DI-2594

Immunocytochemical processing involved fixing cells in 4% paraformaldehyde and subsequently permeabilizing them with Triton X-100 (0.1%) in PBS. Blocking was carried out with 0.1% Tween 20 (PBST) supplemented with 1% BSA (CELLNEST, cat# CNB104-0100). The cells were then incubated overnight at 4°C with the primary antibody. The cells were then incubated for 1 h at room temperature with anti-rabbit DyLight 488 (Vector Laboratories, cat# DI-1488, Burlingame, CA, USA) or anti-mouse DyLight 594 (Vector Laboratories, cat# DI-2594) antibodies. Finally, the cells were nuclear stained with 1 μg/mL DAPI (4^′^,6-diamidino-2-phenylindole) at room temperature for 5 min. Images were obtained using a Nikon confocal microscope (Nikon, Tokyo, Japan).

### 2.3 RNA Interference and Real-Time RT-qPCR (Reverse Transcription-Quantitative Polymerase Chain Reaction

siRNA transfections (0.1 μg) were performed using Oligofectamine (Invitrogen, cat# 12252011, Carlsbad, CA, USA) following the manufacturer’s instructions. The sequences for siRNAs and shRNAs are shown in Table 2. Lentiviral transduction of TamR cells (8 × 10^5^ cells/well, 6-well plate) was performed at an MOI of 10 in the presence of 8 μg/mL polybrene for 20 h using lentiviral vectors expressing either control shRNA or shRNAs targeting SETD1A and FOXA2 (Lugen Sci, Bucheon-si, Republic of Korea), followed by puromycin selection (2 μg/mL) for one week. Total RNA was extracted from TamR cells using TRIzol reagent (Invitrogen, cat# 15596018) and subsequently treated with DNase I (1 U) to remove any residual genomic DNA, and cDNA was generated with the iScript kit. (Bio-Rad Laboratories, cat# 1708891). For real-time quantitative PCR, the reverse transcription products were analyzed using a LightCycler 480 II (Roche, Indianapolis, IN, USA), employing the primers listed in Table 3. Expression values were normalized against 18S rRNA. Quantitative PCR (qPCR) was performed in a 10 μL reaction volume with 0.2 μM of each primer, using the following cycling conditions: initial denaturation at 95°C for 5 min, then 45 cycles of 95°C for 10 s, 60°C for 10 s, and 72°C for 10 s. Target gene concentration in each sample was normalized by dividing it by the concentration of the 18S rRNA reference gene in the corresponding sample. The average normalized expression for each treatment group was then divided by the average normalized expression of the control (blank) group to calculate the Relative Fold Change.

**Table 2:** siRNA and shRNA sequences used in the study

Name	Sequence
shNS	CCGGCAACAAGATGAAGAGCACCAACTCGAGTTGGTGCTCTTCATCTTGTTGTTTTT
shFOXA2 (#1)	CCGGGCAAGGGAGAAGAAATCCATACTCGAG TATGGATTTCTTCTCCCTTGCTTTTTG
shFOXA2 (#2)	CCGGGCAAGGGAGAAGAAATCCATACTCGAGTATGGATTTCTTCTCCCTTGCTTTTTG
shSETD1A (#1)	CCGGCGGAAGAAGAAGCTCCGATTTCTCGAGAAATCGGAGCTTCTTCTTCCGTTTTTTG
shSETD1A (#2)	CCGGCTTTGCGGAGAAGAAGCTGTACTCGAGTACAGCTTCTTCTCCGCAAAGTTTTTTG
siNS	UUCUCCGAACGUGUCACGUdTdT (sense)
	ACGUGACACGUUCGGAGAAdTdT (anti-sense)
siFOXA2 (#1)	GCAAGGGAGAAGAAAUCCAUAdTdT (sense)
	UAUGGAUUUCUUCUCCCUUGCdTdT (anti-sense)
siFOXA2 (#2)	GAACGGCAUGAACACGUACAUdTdT (sense)
	AUGUACGUGUUCAUGCCGUUCdTdT (anti-sense)

Note: FOXA2: Forkhead Box Protein A2; SETD1A: SET Domain Containing 1A.

**Table 3:** Primer sequences for reverse transcription-quantitative polymerase chain reaction, Chromatin IP, and Formaldehyde-assisted isolation of regulatory elements-qPCR

Name	Forward (5^′^-3^′^)	Reverse (5^′^-3^′^)
*FOXA2*	GGAGCAGCTACTATGCAGAGC	CGTGTTCATGCCGTTCATCC
*FOXA1*	GCAATACTCGCCTTACGGCT	TACACACCTTGGTAGTACGCC
*18S*	GAGGATGAGGTGGAACGTGT	TCTTCAGTCGCTCCAGGTCT
*FOXA2 −1kb*	CCTTGGTTTTCACTGCCGTT	TCCCCATCTCCCTCCAAAAC
*FOXA2 −0.5kb*	AGTCCCAATTCCACCACTGT	CACAGCTTGCTTCTCTTGGG
*FOXA2 0kb*	ACACTCCCTGGGCCAAAATA	GTGTATTCTGGCTGCAAGGG
*FOXA2 +0.5kb*	TCACCGTGTCAAGATTGGGA	TCCTCCATTGCTGTTGTTGC
*FOXA2 +1kb*	TCATAATGGGCCGGGAGTAC	ACAACCTCATGTCCTCGGAG

### 2.4 Examination of Newly Synthesized Messenger RNA

Newly transcribed RNA was labeled and purified with a Click-iT Nascent RNA Capture Kit (Invitrogen, cat# C10365). TamR cells (5 × 10^5^ cells/mL) were treated with 0.2 mM 5-ethynyl uridine (EU) for 16 h to label the nascent RNAs. Following labeling, Total RNA was isolated from cells using TRIzol reagent (Invitrogen, cat# 15596018) according to the manufacturer’s instructions and subsequently treated with DNase I (1 U). For the click reaction, 1 μg of RNA was used, and 12 μL of a Dynabeads culture (Invitrogen, cat# 65601) was employed for capturing 1 μg of biotin-conjugated RNA labeled with EU. cDNA was generated using the iScript cDNA Synthesis Kit (Bio-Rad Laboratories, cat# 1708891) with random hexamers as primers in a total reaction volume of 40 μL, following the manufacturer’s instructions. Quantitative PCR (qPCR) was analyzed in a 10 μL reaction volume with 0.2 μM of each primer, using a Roche LightCycler 480 II instrument (Roche), equipped with SYBR Green dye (Roche, cat# 50-720-3180). Target gene concentration in each sample was normalized by dividing it by the concentration of the *18S* internal control gene in the corresponding sample. The average normalized expression for each treatment group was then divided by the average normalized expression of the control (blank) group to calculate the Relative Fold Change.

### 2.5 Chromatin Immunoprecipitation Assay

Chromatin immunoprecipitation (ChIP) assays were performed as previously described [31]. Briefly, Control or FOXA2-silenced TamR cells (1.5 × 10^7^ cells) were cross-linked with 1% formaldehyde for 30 min, followed by 0.125 M glycine to terminate the cross-linking reaction. Chromatin extracts were then prepared, and sonicated chromatin solutions were subjected to immunoprecipitation. This was achieved by incubating samples overnight at 4°C with antibodies against 10 μg SETD1A (Bethyl Laboratories), 2 μg H3K4me3 (Active Motif), 2 μg RNA polymerase II (Merck Millipore), or 2 μg rabbit IgG (Cell Signaling Technology) as a control. precipitated DNA analyzed using a Roche LightCycler 480 II system equipped with SYBR Green dye (Roche, cat# 50-720-3180). The following conditions were used for qPCR amplification: pre-incubation at 95°C for 5 min, then 45 cycles of 95°C for 10 s, 60°C for 10 s, and 72°C for 10 s. Data are shown relative to input chromatin, with ChIP assay primer sequences detailed in Table 3.

### 2.6 Formaldehyde-Assisted Isolation of Regulatory Elements (FAIRE)-qPCR

FAIRE-qPCR was performed as previously described [32]. Results are shown relative to input chromatin, with the FAIRE-qPCR primers listed in Table 3. Control or FOXA2-silenced TamR cells (1.5 × 10^7^ cells) were cross-linked with 1% formaldehyde for 30 min, followed by 0.125 M glycine to terminate the cross-linking reaction. and prepare 2 mL of extract. The cross-linked chromatin was sonicated, and the open chromatin (FAIRE DNA) was purified from the aqueous phase via phenol–chloroform extraction. DNA was then precipitated by adding 100% ethanol and 3 M sodium acetate, followed by incubation at −80°C overnight. For input control (Input DNA), cross-linking was reversed by heating before extraction. The precipitated DNA was collected by centrifugation at 10,000× *g* for 30 min at 4°C, washed twice with 70% ethanol, and finally dissolved in 100 μL of TE buffer and analyzed using a Roche LightCycler 480 II system equipped with SYBR Green dye (Roche, cat# 50-720-3180). qPCR was performed using 1 μL of template in a total reaction volume of 10 μL with the following conditions: initial denaturation at 95°C for 5 min, then 45 cycles of 95°C for 10 s, 60°C for 10 s, and 72°C for 10 s. FAIRE-qPCR enrichment was calculated as the percentage of input.

### 2.7 Cell Proliferation, Migration, and Invasion Assay

Control or FOXA2-silenced TamR cells were cultured in DMEM (Hyclone) supplemented with 10% FBS (Gibco) and 5 μM 4-hydroxytamoxifen. Control or FOXA2-silenced MDA-MB-231 cells were cultured in RPMI-1640 medium (Hyclone, cat# SH30027.01) supplemented with 10% FBS These cells were plated in 24-well plates at a density of 1 × 10^4^ cells per well and incubated at 37°C with 5% CO_2_. The plates were then transferred to an IncuCyte Live Cell HD Imaging system (Essen Bioscience, Ann Arbor, MI, USA). Migration assays were performed using IBIDI culture inserts (IBIDI GmbH, Martinsried, Germany). Control or FOXA2-silenced TamR cells (4 × 10^5^ cells/mL) were seeded into the reservoirs of the inserts and placed in a 24-well plate. Cell migration was monitored using the IncuCyte Live Cell HD Imaging System. Cell invasion was assessed using BioCoat™ Matrigel® Invasion Chamber (354480, Corning Inc., Corning, NY, USA). 100 μL of serum-free DMEM and 200 μL of Control or FOXA2-silenced TamR cells (1.5 × 10^5^ cells/mL) suspended in serum-free DMEM were added to the upper chamber. The invasion assay was carried out for 48 h, with 750 μL of medium supplemented with 20% FBS in the lower chamber serving as a chemoattractant. After staining with 0.1% crystal violet for 15 min and washing with PBS, cells remaining in the upper chamber were removed. Migrated or invaded cells were photographed using a Nikon TS100 stereomicroscope with a Canon G10/G11 camera (Canon, Tokyo, Japan), and at least three random fields per insert were quantified using ImageJ [31].

### 2.8 Mammosphere Formation Assay

Control or FOXA2-overexpresed MCF-7 cells, Control or FOXA2-silenced TamR cells, Control or FOXA2-silenced MDA-MB231 cells (1 × 10^3^ cells/mL, 200 μL per well) were suspended in a mammosphere medium consisting of 20 ng/mL epidermal growth factor, 10 ng/mL basic fibroblast growth factor, 5 μg/mL insulin, 0.4% BSA, B27 Supplement (Gibco, cat# 17504044), and DMEM (Hyclone, cat# SH30243.01) or RPMI-1640 (Hyclone, cat# SH30027.01) medium and plated into 96-well ultra-low attachment plates (Corning, cat# 3474, Corning, NY, USA). The cells were incubated at 37°C using 5% CO_2_ for 7 days. The formation of mammospheres was examined microscopically (ECLIPSE TS100-F, Nikon, Tokyo, Japan) at 10× magnification. The percentage of mammospheres formation = (Number of mammospheres/Number of plated single cells).

### 2.9 Collection and Examination of Transcriptomic Data

The receiver operating characteristic (ROC) Plotter platform (http://rocplt.org) (v.2023) [33] was utilized to assess the prognostic relevance of FOXA2 expression in individuals with BC. Treatment response was determined using reported pathological complete response data (n = 1775) or 5-year recurrence-free survival (n = 1329), and the prognostic significance of FOXA2 levels in breast cancer patients receiving chemotherapy was assessed accordingly. Patients receiving adjuvant therapy were divided into two groups based on their survival status at 5-year follow-up: one group consisted of patients with no histological evidence of residual tumor after chemotherapy (responder), and the other group consisted of all patients with residual tumor tissue (non-responder). The two groups were compared using the R statistical environment (version 4.3.2; www.r-project.org) and the Mann-Whitney U test or ROC curve test from the Bioconductor library (version 3.22; www.bioconductor.org). Statistical significance was set at *p* < 0.05. Data for SETD1A and FOXA2 mRNA expression in tamoxifen-treated breast cancer patients were retrieved from the GSE9893 microarray dataset, which had been log2-transformed. The expression profiles were derived from primary tumor tissues collected from a cohort of 155 patients treated with tamoxifen, all of whom had clinical follow-up data spanning more than five years. The cohort was stratified into relapse and relapse-free groups according to disease progression status. Subsequently, expression data corresponding to the SETD1A and FOXA2 genes were isolated from the overall gene expression profiles of each participant.

### 2.10 Immunofluorescent Analysis of Tissue Microarrays

A human BC tissue microarray (US Biomax, cat# BRM961b, Rockville, MD, USA) comprising paired primary tumors (n = 36) and matched lymph node metastases (n = 36) was analyzed. The sections were incubated with Tris-EDTA buffer at 115°C for 5 min for antigen retrieval. Blocking was performed with a serum-free protein blocking buffer (Agilent Daco, cat# X0909, Santa Clara, CA, USA) at room temperature for 1 h. The samples were treated with primary antibodies against SETD1A (1:100, Santa Cruz Biotechnology, cat# SC-515590) and FOXA2 (1:100, ABclonal Technology, cat# A19053, USA) and incubated at 4°C overnight. Subsequently, the slides were incubated for 20 min at room temperature with DyLight 488-conjugated anti-rabbit and DyLight 594-conjugated anti-mouse secondary antibodies (1:200, Vector Laboratories) (Table 1). Images were acquired via confocal microscopy (Nikon, Tokyo, Japan), and integrated optical density (IOD) values were quantified using ImageJ software (v1.8.0, National Institutes of Health, Bethesda, MD, USA).

Following deparaffinization, sections were incubated with primary antibodies against SETD1A (Santa Cruz Biotechnology, cat# SC-515590) and FOXA2 (ABclonal Technology) overnight at 4°C. Subsequently, the slides were incubated at room temperature for 1 h with DyLight 488-conjugated anti-rabbit and DyLight 594-conjugated anti-mouse secondary antibodies (Vector Laboratories) (Table 1). Images were acquired via confocal microscopy (Nikon, Tokyo, Japan), and integrated optical density (IOD) values were quantified using ImageJ (v1.8.0) (National Institutes of Health, Bethesda, MD, USA).

### 2.11 Statistics

Statistical analyses of cell viability, migration, quantitative PCR (qPCR), and protein correlation data were performed using GraphPad Prism software v8.02 (GraphPad, La Jolla, CA, USA). Data are presented as mean ± standard deviation (S.D.). For comparisons amongst groups, one-way analysis of variance (ANOVA) was performed prior to Dunnett’s post-hoc test to assess differences between each experimental group and the control. *p < 0.05, **p < 0.01, ***p < 0.001. Where appropriate, correlation analyses were performed using Pearson’s correlation coefficient to assess relationships between different protein expression levels.

## 3 Results

### 3.1 FOXA2 Is Overexpressed in TamR BC Cells

**Figure 1:**
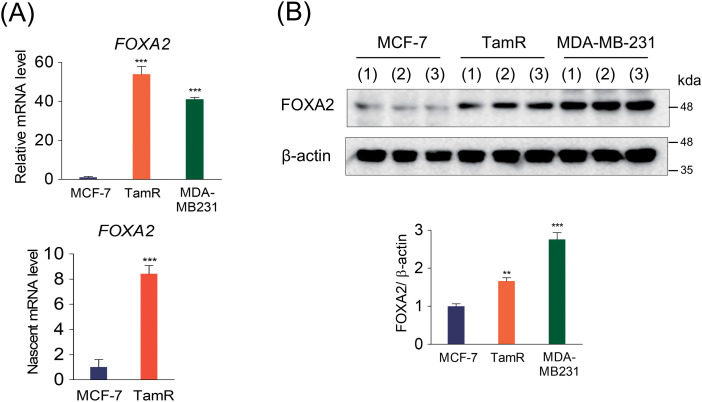

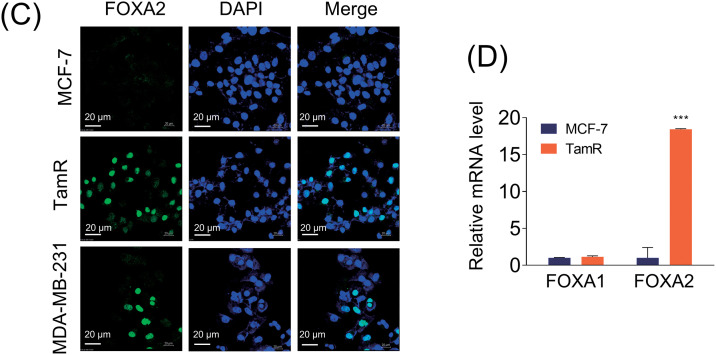
Increased FOXA2 expression in tamoxifen-resistant MCF-7 (TamR) and MDA-MB-231 cells. (**A**) mRNA (left panel) and nascent mRNA (right panel) levels of FOXA2 were compared among parental breast cancer cells (MCF-7), their corresponding tamoxifen-resistant MCF-7 cells (TamR), and MDA-MB-231 cells. FOXA2 expression was quantified using RT-qPCR to assess relative mRNA levels (n = 3). (**B**) Comparison of FOXA2 protein levels in MCF-7, TamR, and MDA-MB-231 cells measured using western immunoblotting (n = 3). Densitometric analysis of FOXA2 protein levels normalized to β-actin loading control in MCF-7, TamR, and MDA-MB-231 cells. (**C**) Immunofluorescence analysis of FOXA2 (green) levels in MCF-7, TamR, and MDA-MB-231 cells. (**D**) Comparison of FOXA1 and FOXA2 mRNA levels in MCF-7 and TamR cells (n = 3). **p < 0.01; ***p < 0.001

From a previous transcriptome analysis, we identified that FOXA2 is a target gene of SETD1A and that SETD1A expression is increased in TamR cells [31]. Therefore, we compared the mRNA, nascent mRNA, and protein levels of FOXA2 in MCF-7 cells, TamR cells, and ERα-negative BC MDA-MB-231 cells (Fig. 1A,B). FOXA2 expression was significantly higher in TamR and MDA-MB-231 cells than in MCF-7 cells. Immunofluorescence analysis of endogenous FOXA2 revealed that FOXA2 was undetectable in MCF-7 cells but abundant in TamR and MDA-MB-231 cells (Fig. 1C). Significant overexpression of FOXA2 in the TNBC cell line MDA-MB-231 represents the FOXA2 in TamR BC. The expression levels of FOXA1, another member of the FOXA transcription factor family and an important regulator of ER-mediated transcription, were almost unchanged in MCF-7 and TamR cells (Fig. 1D). These results suggest that FOXA2 is involved in tamoxifen resistance in BC.

### 3.2 SETD1A Regulates the Transcription of FOXA2

**Figure 2:**
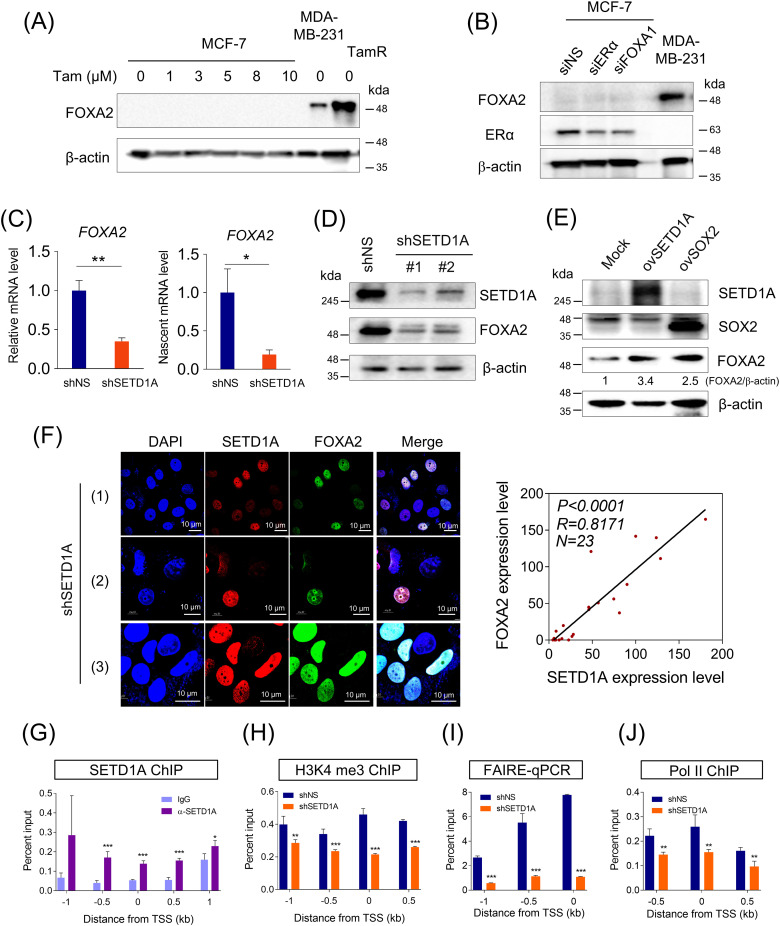
SET Domain Containing 1A (SETD1A) regulates the expression of the FOXA2 gene. (**A**) MCF-7 cells were treated with different concentrations of tamoxifen for 24 h, and FOXA2 levels were assessed by western blotting analysis. β-Actin was used as the loading control. The FOXA2 levels in MDA-MB-231 and TamR cells were used as a positive control. (**B**) ERα or FOXA1 was knocked down in MCF-7 cells. ERα and FOXA2 levels were assessed by western blotting analysis. β-Actin was used as the loading control. (**C**) Analysis of mRNA and nascent mRNA levels of FOXA2 in SETD1A-depleted TamR cells. (**D**) Effects of SETD1A on FOXA2 protein expression. Protein levels of SETD1A and FOXA2 in TamR cells depleted of SETD1A using two independent shRNAs were evaluated using western blotting, with β-actin serving as the loading control. (**E**) Overexpression of SETD1A (ovSETD1A) or SOX2 (ovSOX2) in MCF-7 cells and FOXA2 levels were assessed by western blotting analysis. β-Actin was used as the loading control. (**F**) SETD1A (red) and FOXA2 (green) immunofluorescence in TamR cells transfected with shSETD1A (n = 3). Fluorescence intensities are plotted in the right panel, and the correlation between SETD1A and FOXA2 expression was assessed via Pearson analysis using GraphPad Prism v.8.0.2 (n = 23). (**G**) ChIP assays were performed in TamR cells to examine SETD1A recruitment at the FOXA2 locus. Quantification of the precipitated FOXA2 region by anti-SETD1A antibody was conducted via quantitative PCR. (**H**) Investigation of SETD1A’s role in H3K4me3 methylation at the FOXA2 locus. SETD1A expression was knocked down in TamR cells using shRNA. (**I**) Investigation of SETD1A-mediated chromatin accessibility at the FOXA2 gene via formaldehyde-assisted isolation of regulatory elements (FAIRE)-qPCR. (**J**) Effects of SETD1A depletion on Pol II binding to the FOXA2 promoter (n = 3). *p < 0.05; **p < 0.01; ***p < 0.001

Since TamR cells acquired resistance after MCF-7 cells were treated with low tamoxifen concentrations, we aimed to determine whether tamoxifen treatment increased FOXA2 expression in TamR cells. Tamoxifen did not appear to directly influence FOXA2 expression (Fig. 2A). Reduced ERα or FOXA1 expression is characteristic of tamoxifen resistance and may contribute to an increase in FOXA2 expression. Accordingly, we examined whether ERα and FOXA1 protein levels influence FOXA2 expression. Depletion of ERα or FOXA1 did not increase FOXA2 expression (Fig. 2B). However, we found that SETD1A, a histone H3K4 methyltransferase, was critical for FOXA2 transcription. SETD1A knockdown resulted in the downregulation of mRNA, nascent mRNA, and protein levels of FOXA2 (Fig. 2C,D). Conversely, the overexpression of SETD1A or SOX2 increased FOXA2 expression (Fig. 2E). SOX2 is a regulator of the FOXA2 gene [34] and a partner of SETD1A in TamR cells [31]. Immunofluorescence detection of endogenous SETD1A and FOXA2 revealed that the levels of SETD1A and FOXA2 proteins in the nucleus were correlated. TamR cells exhibiting reduced SETD1A levels also showed decreased FOXA2 expression, and the converse was true (Fig. 2F). These results suggest that SETD1A directly modulates the transcription of FOXA2. To investigate the mechanism of FOXA2 transcription regulation by SETD1A, we examined the occupancy of SETD1A to the FOXA2 gene in TamR cells. Chromatin immunoprecipitation (ChIP) analysis revealed that the promoter region of FOXA2 was occupied by SETD1A (Fig. 2G). SETD1A knockdown using shRNA reduced H3K4me3 occupancy, chromatin accessibility, and Pol II binding to the FOXA2 gene promoter (Fig. 2H,I).

### 3.3 FOXA2 Promotes the Proliferation and Migration of TamR

**Figure 3:**
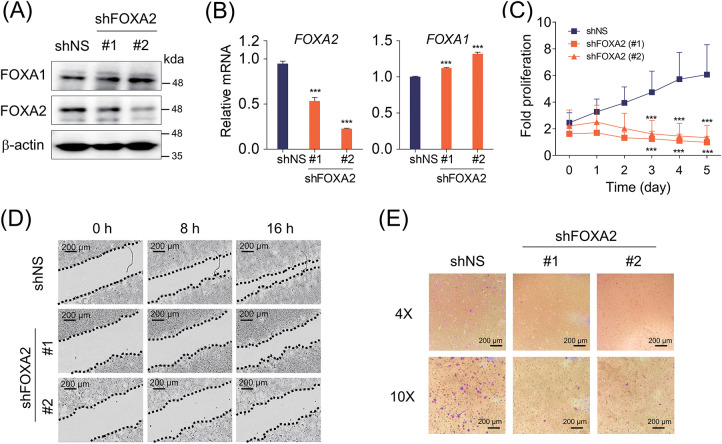

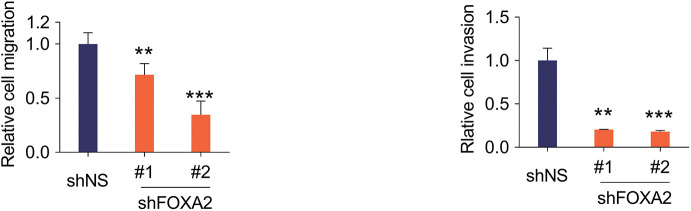
FOXA2 promotes the proliferation and migration of TamR cells. (**A**) Protein levels of FOXA2 and FOXA1 in TamR cells depleted of FOXA2 using two independent shRNAs were evaluated using western blotting, with β-actin serving as the loading control. (**B**) FOXA2 was knocked down using shRNA in TamR cells, and the mRNA levels of FOXA1 and FOXA2 were assessed. (**C**) Cell proliferation in FOXA2-depleted TamR cells was monitored via live-cell imaging. Data are presented as mean ± S.D. (n = 6). (**D**) Cell migration assay of FOXA2-depleted TamR cells. The migration of TamR cells was evaluated after 8 and 16 h. The top panel displays representative images, and the bottom panel illustrates quantification based on the distance between opposing cell boundaries (n = 3). (**E**) Transwell invasion assays of FOXA2-depleted TamR cells. Shown in the top panel are representative images illustrating cell invasion. Cell invasion was quantified by counting the number of invaded cells per field under a microscope (bottom panel) (n = 3). **p < 0.01; ***p < 0.001

**Figure A1:**
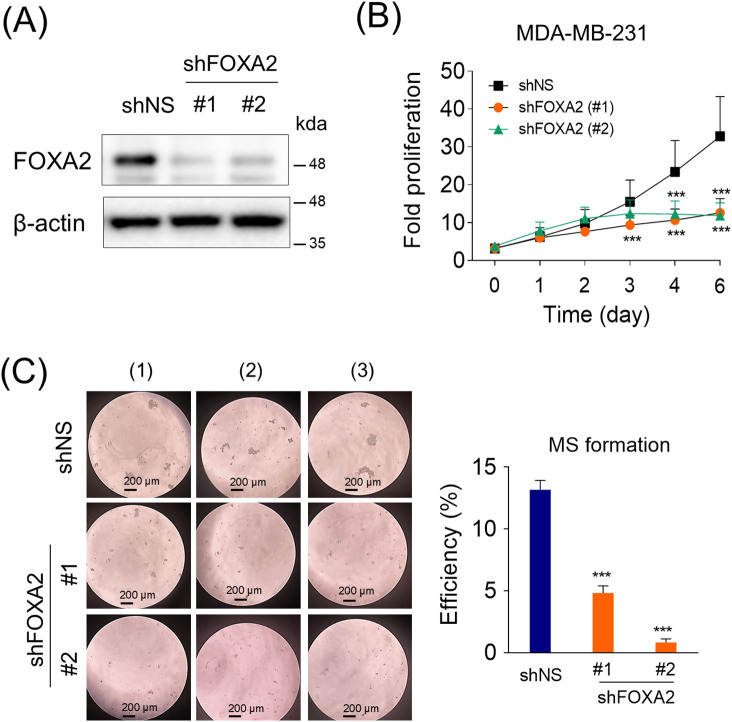
Role of forkhead box protein A2 (FOXA2) in MDA-MB231 cells. (**A**) FOXA2 was depleted using shRNA in MDA-MB-231 cells, and the protein levels of FOXA2 were assessed. (**B**) Cell proliferation in FOXA2-silenced MDA-MB231 was evaluated using live-cell imaging (n = 6). (**C**) Mammosphere formation assay of FOXA2-depleted MDA-MB-231 cells. Mammosphere formation efficiency was assessed as the ratio of formed mammospheres to the number of plated cells (n = 3). ***p < 0.001

The increase in FOXA2 protein levels mediated by SETD1A in TamR cells prompted investigation into the functional significance of FOXA2 in this context. First, we knocked down endogenous FOXA2 in TamR cells using shRNA, and this manipulation did not affect FOXA1 protein and mRNA levels (Fig. 3A,B). We then measured changes in cell proliferation owing to FOXA2 depletion. Growth of TamR and MDA-MB-231 cells was significantly inhibited by FOXA2 depletion (Figs. 3C and A1). Additionally, FOXA2 depletion significantly inhibited TamR cell migration and invasion (Fig. 3D,E). The results imply that FOXA2 contributes significantly to the proliferation and migration of TamR BC cells.

### 3.4 FOXA2 Induces Sphere Formation and Tamoxifen Resistance in BC Cells

**Figure 4:**
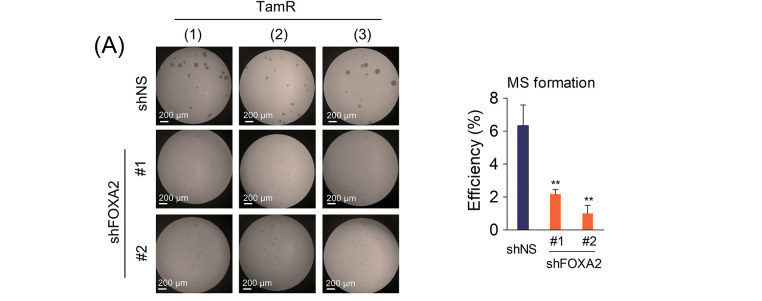

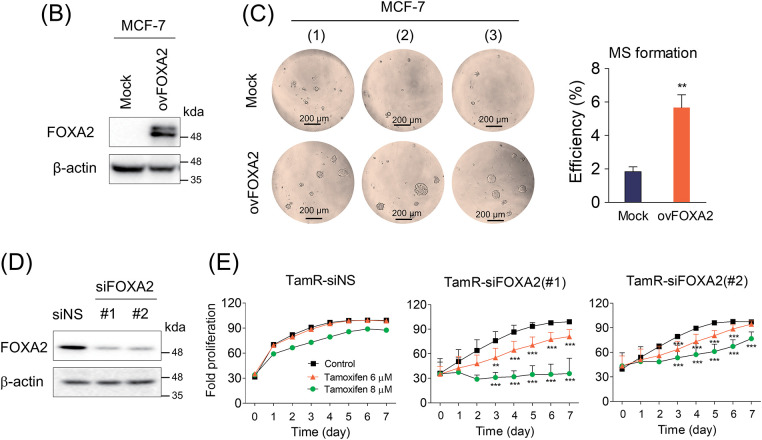
FOXA2 induces sphere formation and tamoxifen resistance in breast cancer cells. (**A**) Mammosphere formation assay of FOXA2-depleted TamR cells. Mammosphere formation efficiency was assessed as the ratio of formed mammospheres to the number of plated cells (n = 3). (**B**) FOXA2 was overexpressed in MCF-7 cells by transfection with a FOXA2 expression plasmid (ovFOXA2). (**C**) The effect of FOXA2 overexpression on mammosphere formation in MCF-7 cells was assessed. Mammosphere formation was assessed in MCF-7 cells following transfection with either control or FOXA2-overexpressing plasmids (mock vs. ovFOXA2) (n = 3). (**D**) FOXA2 was depleted in MCF-7 cells using two independent siRNAs. (**E**) Effects of FOXA2 knockdown on tamoxifen sensitivity in TamR cells. Control (TamR-siNS) and FOXA2-depleted (TamR-siFOXA2) cells were cultured with tamoxifen, and proliferation was monitored via live-cell imaging. **p < 0.01; ***p < 0.001

SETD1A contributes to CSC maintenance of cancer stem cells by regulating SOX2 [31]. In addition, FOXA2 was found to be a target gene of both SETD1A and SOX2 (Fig. 2D,E). Based on these findings, we investigated the effects of FOXA2 on mammosphere formation. FOXA2 depletion led to a significant reduction in mammosphere formation in TamR cells (Fig. 4A). Conversely, when FOXA2 was overexpressed in MCF-7 cells, increased mammosphere formation was observed (Fig. 4B,C). These results not only indicate the importance of FOXA2 in regulating cancer stem cell properties but also support its involvement in tamoxifen resistance mechanisms. To confirm the role of FOXA2 in tamoxifen resistance, we examined the effect of FOXA2 on tamoxifen sensitivity in BC cells. Compared to the control group (TamR-siNS), FOXA2-depleted TamR cells (TamR-siFOXA2) showed significant restoration of sensitivity to tamoxifen (Fig. 4D,E). These results demonstrated the critical role of FOXA2 in the development of tamoxifen resistance in BC cells and suggested that tamoxifen sensitivity may be modulated by FOXA2 regulation.

### 3.5 Clinical Significance of FOXA2 in Patients with BC

**Figure 5:**
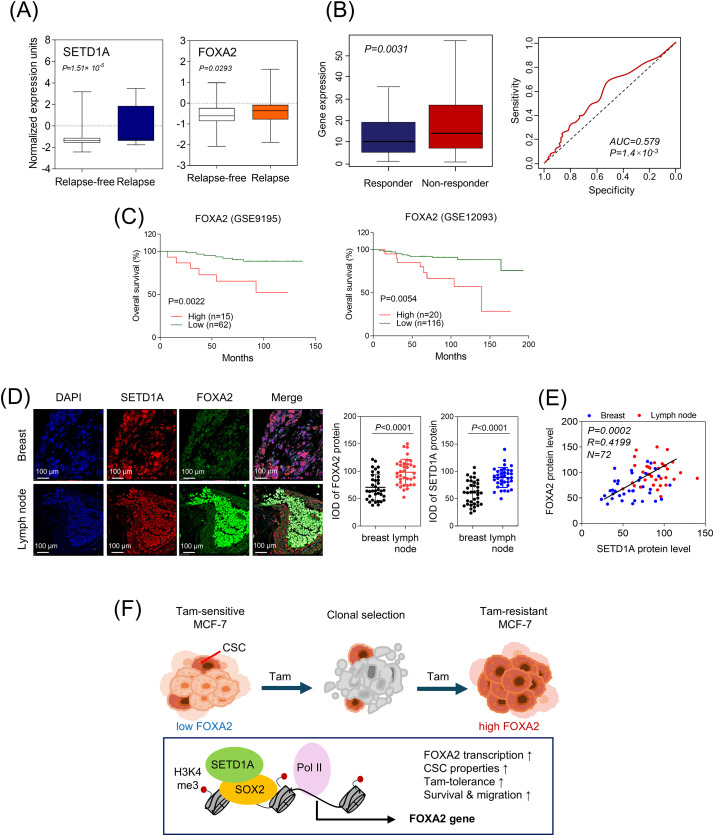
Clinical significance of FOXA2 in patients with tamoxifen-resistant breast cancer. (**A**) mRNA expression of SETD1A and FOXA2 in ERα-positive breast cancer patients treated with tamoxifen (GSE9893). The closed box indicates values between the 25th and 75th percentiles. (**B**) FOXA2 expression analysis predicts poor outcomes in patients with breast cancer treated with chemotherapy. The area under the curve (AUC) and corresponding *p*-value were determined for the FOXA2 gene signature. (**C**) Kaplan–Meier overall survival curves illustrate the prognostic significance of FOXA2 signature. Patients with FOXA2 expression above the cohort median were classified as the high-expression group, while the rest were designated as the low-expression group. (**D**) Representative images depict SETD1A and FOXA2 expressions in breast cancer tissue samples. Immunofluorescence staining was performed on tissue microarrays of malignant human breast cancer samples (breast, n = 36) and matched metastatic lymph node tissues (lymph node, n = 36). Protein expression levels for each sample were quantified as integrated optical density (IOD). (**E**) Correlation between SETD1A and FOXA2 expression in breast cancer tissues was evaluated using scatter plots of quantified protein levels in malignant breast and lymph node tissues. Pearson’s correlation coefficient was calculated using GraphPad Prism v8.0.2. (**F)** Proposed mechanism by which FOXA2 acts as a SETD1A-regulated driver of tamoxifen resistance in breast cancer

Finally, the prognostic relevance of SETD1A and FOXA2 expression was assessed in tamoxifen-resistant breast cancer patients following chemotherapy. Analysis of the transcriptome in ER-positive BC patients treated with tamoxifen (GSE9893) [35] revealed that the transcription levels of both SETD1A and FOXA2 were significantly higher in TamR patients than in those who remained sensitive to tamoxifen (Fig. 5A). Moreover, FOXA2 expression was significantly higher in patients who did not respond to chemotherapy (*p* = 0.0031) and the AUC value was 0.579, indicating that FOXA2 may have potential clinical utility as a cancer biomarker (Fig. 5B). In the cohort of patients with ERα-positive BC treated with tamoxifen (GSE9195 and 12093) [36,37], a strong correlation was observed between elevated FOXA2 mRNA levels and decreased overall survival (Fig. 5C). The protein expression of SETD1A and FOXA2 in patients with metastatic BC was markedly higher in metastatic lymph node tissues (lymph node) than in malignant breast tissues (breast, Fig. 5D). Furthermore, their expression levels exhibited a significant positive correlation in patients with BC (Fig. 5E). These findings suggest that FOXA2 could be a valuable biomarker for identifying patients at higher risk of tamoxifen resistance and poor prognosis, potentially guiding personalized treatment strategies for BC management (Fig. 5F).

## 4 Discussion

Endocrine therapy plays a crucial role in lowering mortality and recurrence rates in patients with ERα-positive BC by targeting and inhibiting ERα signaling. This approach has significantly enhanced survival rates and reduced the likelihood of recurrence. Nevertheless, over 30% of ER-positive BC cases exhibit intrinsic resistance to hormone therapy from the outset [38]. In addition, resistance to long-term endocrine therapy often develops over time, leading to acquired resistance [39]. Our study suggests a novel molecular mechanism that drives tamoxifen resistance in BC.

FOXA2 is a transcription factor belonging to the forkhead/winged-helix family that plays a dual role in cancer biology. It has been implicated in suppressing tumor growth in cervical cancer and mitigating epithelial-mesenchymal transition (EMT) in hepatocellular carcinoma (HCC), thereby controlling metastasis [40]. FOXA2 also acts as an oncogene that promotes tumorigenesis. For instance, FOXA2 is a key driver of NEPC and facilitates stemness and tumor sphere formation in pancreatic cancer stem cells [19]. FOXA2 is an oncogene in CRC that promotes EMT and metastasis [17]. EMT, a process by which epithelial cells acquire a mesenchymal phenotype, plays a pivotal role in therapeutic resistance and tumor recurrence. FOXA2 is also associated with the inhibition of EMT in BC [41].

In this study, we found that TamR BC cells exhibited significant upregulation of FOXA2, a transcription factor implicated in the regulation of diverse cellular processes. Furthermore, we elucidated the mechanism by which FOXA2 levels significantly increased in TamR cells. To investigate the reason for the significant increase in FOXA2 levels in TamR cells, we examined whether it could be attributed to tamoxifen or ERα influences. The results showed that neither tamoxifen nor ER affected FOXA2 expression. Based on our previous transcriptome analysis, we hypothesized that FOXA2 was a target of SETD1A. SETD1A knockdown directly decreased the levels of nascent FOXA2 mRNA, and SETD1A was found to bind to the promoter region of the FOXA2 gene. Conversely, SETD1A overexpression increased FOXA2 protein levels. Immunofluorescence revealed a positive correlation between SETD1A and FOXA2 expression in TamR cells. In addition, we showed that elevated FOXA2 levels were negatively associated with several therapeutic aspects of BC, including increased cell proliferation, invasiveness, and the proportion of CSC-like populations. Notably, CSCs are inherently resistant to anticancer therapies, which is a major obstacle to effective treatment [42,43].

This study is to identify FOXA2 as a critical regulatory factor in BC, particularly in TamR BC. Although FOXA1 has been well characterized as a pioneer factor essential for ERα signaling and maintenance of endocrine therapy responsiveness, the role of FOXA2 has remained relatively underexplored. Our findings highlight that FOXA2 contributed to tamoxifen resistance through an independent transcriptional regulatory mechanism distinct from FOXA1 and was transcriptionally regulated by SETD1A, underscoring the significance of this novel regulatory axis. Moreover, we demonstrate that FOXA2 was closely associated with the enhancement of cancer stem cell properties, increased cell proliferation, and invasiveness, revealing its functional contribution to BC therapeutic resistance and pathological progression that are distinct from those of FOXA1. Consequently, FOXA2 is a potential therapeutic target and prognostic biomarker candidate for overcoming endocrine therapy resistance in BC. Importantly, elucidating the SETD1A-FOXA2 axis provides a foundational basis for future studies aimed at developing innovative therapeutics targeting this pathway and establishing personalized treatment strategies. This discovery offers critical insights toward addressing the clinical challenge posed by tamoxifen resistance.

A limitation of this study is that the functional role of FOXA2 as a therapeutic target for overcoming endocrine therapy resistance was not validated across diverse ERα-positive breast cancer subtypes. Further studies are needed to determine whether targeting the SETD1A-FOXA2 axis can be broadly applied in different patient populations.

## 5 Conclusion

This study demonstrates that FOXA2 is a critical regulator of tamoxifen resistance in BC, acting through a transcriptional mechanism controlled by SETD1A. FOXA2 promoted cancer stem cell characteristics, cell proliferation, and invasiveness, highlighting its potential as a therapeutic target and prognostic biomarker. The identification of the SETD1A-FOXA2 axis provides new opportunities for developing targeted therapies and personalized treatment strategies to overcome tamoxifen resistance in BC.

## Data Availability

The data supporting the findings of this study are available from the corresponding authors (M. R. Kim and K. W. Jeong) upon reasonable request.
